# Endothelium and the effect of activated neutrophils on arterial smooth muscle

**DOI:** 10.1515/intox-2015-0008

**Published:** 2015-03

**Authors:** Viktor Bauer, Ruzena Sotnikova

**Affiliations:** Institute of Experimental Pharmacology and Toxicology, Slovak Academy of Sciences, Dúbravská cesta 9, 841 04 Bratislava, Slovak Republic

**Keywords:** rat thoracic aorta, endothelium, nitric oxide, activated neutrophils, hypochlorous acid, peroxynitrite

## Abstract

The aim of the study was to analyze the involvement of the endothelium in the effects of neutrophils (PMNL) on phenylephrine-precontracted isolated rings of the rat thoracic aorta and to compare their effects with those of peroxynitrite (ONOO^−^) and hypochlorous acid (HOCl). Activated PMNL-induced contraction of the precontracted aorta was prevented by the blockade of NO-synthase and by endothelium removal. In the endothelium-free preparations, the effect of PMNL reappeared in the presence of sodium nitroprusside. The effect of ONOO^–^ and HOCl significantly differed from that of activated PMNL both in the presence and absence of the endothelium. It is therefore likely that neither ONOO^–^ nor HOCl generated by transformation of superoxide anion radical (O_2_^•–^) produced by PMNL is involved in their action. Reduction of the relaxant effect of nitric oxide derived from the endothelium by O_2_^•–^ seems to be the keystone mechanism in generation of PMNL-induced contraction.

## Introduction

Maugeri *et al*. (2009) have suggested that processes which activate macrophages in the vessel wall are responsible for manifestations of vascular injury. Endothelial cells play a critical role in the progression of inflammation, atherosclerosis, and tumor angiogenesis. Local inflammation and respiratory burst of polymorphonuclear leukocytes – neutrophils (PMNL), *e.g.* their activation, leads to production and release of superoxide anion radical (O_2_^•–^) (Sand *et al*., 2003; Bauer *et al*., 2008; Jančinová *et al*., 2011). Reactive oxygen species (ROS) diffuse to the vessel wall and the endothelium is their first injured local target. This might be at least in part responsible for the development of altered vessel resistance (Csaki *et al*., 1991; Akopov *et al*., 1992; Tsao *et al*., 1992; De Kimpe *et al*., 1993; Bauer *et al*., 2011a, 2011b).

Our recent findings (Bauer *et al*., 2011a, 2011b) demonstrated the contractile effect of activated PMNL on phenylephrine (PhE)-precontracted rings of the large conduit artery (rat thoracic aorta). Elimination of activated PMNL-induced contractions and of chemiluminiscence by superoxide dismutase suggest that the primarily activated PMNL-released ROS was O_2_^•–^, which can be transformed to other ROS. However, the effect of activated PMNL differed from the action of H_2_O_2_ and OH•. Other ROS which could be involved in the effect of activated PMNL are peroxynitrite (ONOO^–^) and hypochlorous acid (HOCl).

The aim of the present study was to analyze the role of the endothelium in the effect of N-formyl-L-methionyl-L-leucyl-L-phenylalanine (FMLP)-activated peritoneal PMNL on the rat thoracic aorta and to compare it with the effect of ONOO^–^ and HOCl.

## Materials and methods

The study was approved by the Ethics Committee of the Institute and by the State Veterinary and Food Administration of the Slovak Republic. It was performed in accordance with the Principles of Laboratory Animal Care (NIH publication 83-25, revised 1985) and the Slovak law regulating animal experiments (Decree 289, Part 139, July 9^th^ 2003). The guinea-pigs and rats used in the study were purchased from the breeding station of the Institute of Experimental Pharmacology and Toxicology SAS Dobrá Voda, Slovak Republic. The animals were killed by cervical dislocation and exsanguination.

### Chemicals

Phenylephrine hydrochloride, glycogen, N-formyl-L-methionyl-L-leucyl-L-phenylalanine, N_ω_-nitroL-arginine methyl ester (L-NAME), sodium nitroprusside (SNP), percoll, sodium citrate tribasic dehydrate, 5-amino-2,3-dihydro-1,4-phthalazinedione (luminol), trypan blue and NaOCl for preparation of HOCl were all from Sigma-Aldrich Chemie (Steinheim, Germany), ONOO^–^ from Calbiochem (La Jolla, CA) and the other chemicals were of p.a. purity purchased from Lachema (Brno, Czech Republic).

### Solutions

Phosphate buffer saline A *(PBA)* contained (in mmol/l): NaCl 136, KCl 2.6, Na_2_HPO_4_ 8.0, KH_2_PO_4_ 1.5, pH 7.4; phosphate buffer saline B (PBB): NaCl 136, KCl 2.6, Na_2_HPO_4_ 8.0, KH_2_PO_4_ 1.5, CaCl_2_ 0.6, MgCl_2_ 0.5 and glucose 5.6, pH 7.4; and the physiological salt solution *(PSS):* NaCl 112.0, KCl 5.0, KH_2_PO_4_ 1.0, MgSO_4_ 1.2, CaCl_2_ 2.5, NaHCO_3_ 25.0 and glucose 11, pH 7.4. The composition of high potassium depolarizing salt solution *(DSS)* was the same as that of *PSS,* with the only difference that the concentration of KCl was increased with equimolar reduction of NaCl concentration to achieve 100 mmol/l of KCl.

The solution of ONOO^–^ was prepared by dilution in 1 mol/l NaOH. HOCl was diluted in distilled water.

### Isolation and preparation of PMNL

PMNL were isolated from the peritoneal exudate of male Trick guinea-pigs (450–600 g body weight). After intraperitoneal injection of 20 ml of 1.2% glycogen in 0.9% NaCl, the animals were sacrificed within 14 to 16 h. The abdomen was gently massaged after injection of 20 ml of 0.4% trisodium citrate in 0.867% NaCl into the peritoneal cavity and the peritoneal exudate was collected and filtrated. All the following procedures were performed at 4 °C. Cell suspension of the peritoneal exudate was washed with *PBA* and centrifuged for 90 s at 2500 rpm. Erythrocytes were removed by hypotonic treatment. The PMNL pellets were resuspended in *PBA* and 2 ml of their suspension was layered on the discontinuous density gradient of Percoll (1.5 ml of 1.095 g/ml and 1.5 ml of 1.077 g/ml). After centrifugation on the Percoll density gradient for 10 min at 2500 rpm, PMNL were collected from the interface and washed with *PBA* and centrifuged two times for 90 s at 2000 rpm. The PMNL resuspended in *PBA* were counted using Coulter Counter Electronics (England), their viability was assessed using trypan blue and the activity was confirmed also by the ability of ROS production (Sugioka *et al*., 1986; Nosál *et al*., 2002). FMLP induced oxidative burst of PMNL was measured by luminol-enhanced chemiluminiscence in 1 ml samples containing 200 μl isolated PMNL (10^6^/ml), 20 μl luminol (final concentration: 5 μmol/l), 770 μl *PBB* and 10 μl FMLP (final concentration: 0.1 μmol/l) (Drábiková *et al*., 2007; Bauer *et al*., 2011a). PMNL stored in *PBA* stock solutions at 4 °C were used for experiments within the following 2–5 hours.

The low chemiluminiscence of native isolated PMNL, recorded on the basis of luminol-enhanced chemiluminiscence, indicated that the isolation procedure was gentle enough to prevent activation and damage of the isolated PMNL. Upon their activation by introduction of FMLP, a significant respiratory (oxidative) burst developed, evidenced by increase of chemiluminiscence (Bauer *et al*., 2011a).

### Isolation and preparation of rat thoracic aorta

The thoracic aorta removed from male Wistar rats (250–300 g body weight) was immersed in *PSS*. Adherent tissues were cleared and 2 mm long rings were cut. Care was taken not to damage the endothelium. The endothelium-free rings were prepared by denudation of preparations with wet cotton.

The rings were mounted between two platinum hooks. The tissue chamber contained *PSS,* bubbled with a mixture of 95% O_2_ and 5% CO_2_ (pH 7.4) at 37 °C (Sotníková *et al*., 1994). The rings were stretched passively to the resting tension of 20 mN and were allowed to equilibrate for 1 h. During this period of time, *PSS* was repeatedly replaced. The isometric tension was recorded using a strain gauge transducer (Experimetria, Budapest, Hungary).

Experimental protocol: After the equilibration period, the control contraction was induced by *DSS.* The rings were washed 3 times with PSS during 30 minutes until the tension reached initial values. Then PhE in the concentration of 0.3 μmol/l was applied. At the plateau of the contraction, the effect of FMLP (1 μmol/l) and PMNL (10^6^) was recorded. The same protocol was used in the case of denuded preparations and in preparations incubated with L-NAME (0.5 mmol/l). In a different series of experiments, ONOO^–^ in the concentrations of 8 or 160 μmol/l or HOCl in the concentration of 600 μmol/l were applied at the plateau of the PhE-induced contraction. SNP was used in the concentration of 1 nmol/l.

### Analysis of data

All values are given as mean ± SEM of at least 5–7 experiments. Statistical analyses were performed by using two-way analysis of variance with Bonferroni post test. Statistical significance was indicated at p-value less than 0.05.

The mechanical responses are expressed generally as percentages of the PhE- or *DSS-* induced contraction measured at the beginning of each experiment.

## Results

**Figure 1:**
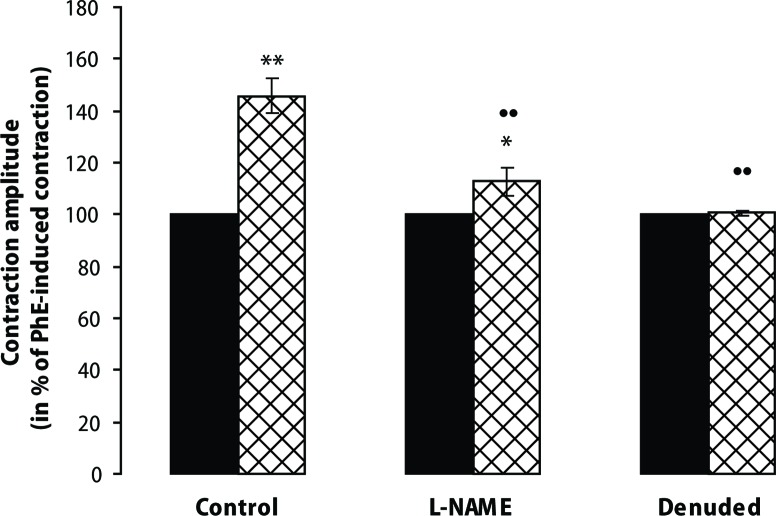
Contraction amplitude of phenylephrine (PhE – 0.3 μmol/l; solid columns)-precontracted isolated rat aortal rings evoked by N-formyl-L-methionyl-L-leucyl-L-phenylalanine (FMLP - 0.1 μmol/l)-activated neutrophils (+PMNL - 10^6^/ml; cross-hatched column) in the absence (Control) and presence of L-NAME (0.5 mmol/l) and after endothelium removal (Denuded). Results are mean ± S.E.M. of 5–7 experiments. *p<0.05, ** *p*<0.01 PMNL *vs* PhE; •• *p*<0.01 control PMNL *vs* denuded or L-NAME treated.

Native PMNL themselves did not alter the level of the contraction elicited by PhE on rings of the rat aorta. When activated with FMLP, however, they elevated the muscle tension significantly by 40–60% of the PhE-induced contraction amplitude. The contraction elicited by activated PMNL was attenuated by L-NAME and was absent in preparations with denuded endothelium (Figure 1).

**Figure 2:**
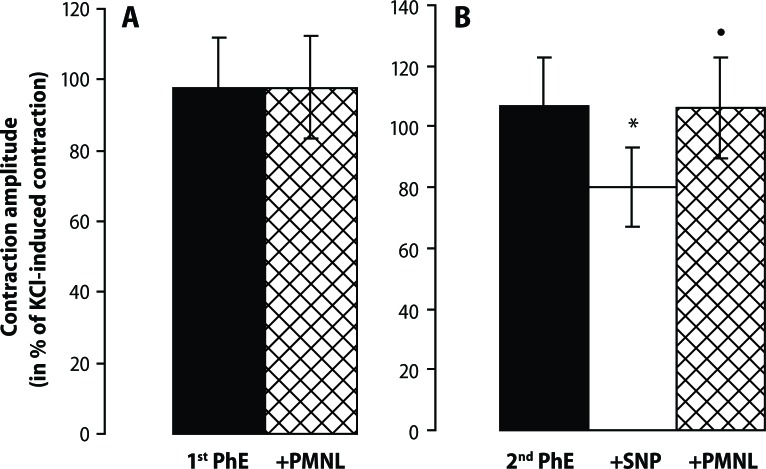
Effect of N-formyl-L-methionyl-L-leucyl-L-phenylalanine (FMLP - 0.1 μmol/l)-activated neutrophils (PMNL - 10^6^/ml; cross-hatched column) in phenylephrine (PhE – 0.3 μmol/l; solid columns)-precontracted denuded rings of the thoracic aorta without (**A**) and in the presence of sodium nitroprusside alone (SNP 1 nmol/l; open column) and SNP with PMNL; cross-hatched column) (**B**). Results are mean ± S.E.M. of 5–7 experiments. •*p*<0.05, *vs* 2^nd^ PhE; *p<0.05 PMNL in untreated *vs* SNP treated.

In the denuded preparations, the activated PMNL were ineffective and SNP relaxed these tissues. Activated PMNL in the presence of SNP elevated the muscle tension of the PhE-precontracted rat aorta (Figure 2).

**Figure 3:**
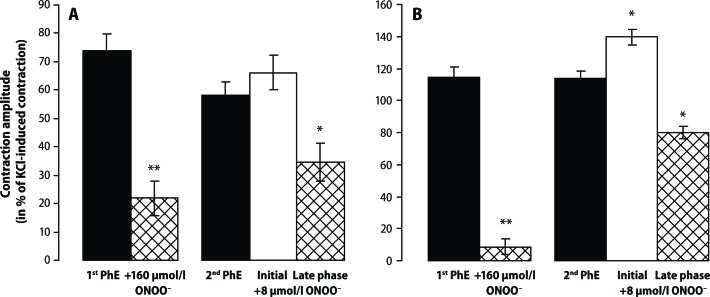
Effect of peroxynitrite (ONOO^–^ – 8 and 160 μmol/l; cross-hatched and open columns) in phenylephrine (PhE – 0.3 μmol/l; solid columns) –precontracted rings of the rat thoracic aorta in the presence (**A**) and absence (**B**) of the endothelium. Results are mean ± S.E.M. of 5–7 experiments. *p<0.05, ** *p*<0.01 ONOO^–^ vs PhE.

Both in the presence and absence of endothelium, ONOO^–^ in a high concentration (160 μmol/l) reduced the PhE-induced contraction of the rat aorta, whereas in low concentration (8 μmol/l) it induced a biphasic response (contraction-relaxation). In the absence of the endothelium, treatment and washout of the high ONOO^–^ concentration did not influence the amplitude of the second applied PhE. On the contrary, in the presence of the endothelium, ONOO^–^ reduced the second PhE contraction amplitude (Figure 3).

**Figure 4:**
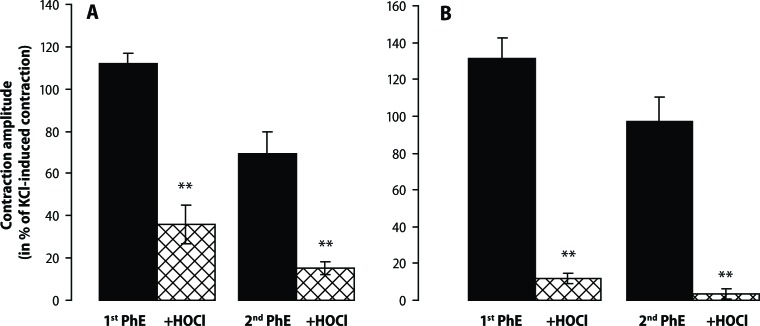
Effect of hypochlorous acid (HOCl – 600 μmol/l; open columns) in phenylephrine (PhE – 0.3 μmol/l; solid columns) – precontracted rings of the rat thoracic aorta in the presence (**A**) and absence (**B**) of the endothelium. Results are mean ± S.E.M. of 5–7 experiments. ** *p*<0.01 HOCl vs PhE.

HOCl markedly reduced the PhE-elevated muscle tension in the presence and practically eliminated it in the absence of the endothelium. This effect was longlasting. After repeated washouts of HOCl, the second application of PhE evoked a significantly smaller contraction compared to that of its first application (Figure 4).

## Discussion

PMNL are considered to be central cells of acute inflammation. These cells most rapidly reach the site of injury or infection and liberate antimicrobial proteins and proteases and produce ROS both intra-and extracellularly. ROS might affect not only the resistance arteries but also the large conduit arteries (Bauer *et al*., 2011a, 2011b). In the rings of the rat thoracic aorta, FMLP-activated PMNL markedly raised the PhE-elevated muscle tension. The elimination of activated PMNL-induced contractions and reduction of FMLP-activated PMNL-induced chemiluminiscence by superoxide dismutase suggest that O_2_^•–^ is the primarily released ROS from activated PMNL, which may eventually be transformed to other ROS (Bauer *et al*., 2011a). These are however not H_2_O_2_ and OH• because their effects differed from that of the activated PMNL (Bauer *et al*., 2011a, 2011b). The other ROS supposed to contribute significantly to tissue damage are ONOO^–^ and HOCl. Since the action of activated PMNL diametrically differs from that of ONOO^–^ and HOCl, their involvement is unlikely. The fact that endothelium removal and blockade of NO synthase by L-NAME attenuated the contractile effect of activated PMNL and their effect reappeared on denuded tissues in the presence of SNP, supports the assumption that O_2_^•–^ interacts with nitric oxide (NO•) continuously released from the endothelium, resulting in elimination of its relaxing potential.

## Conclusion

In the enlargement of the vessel tone by activated PMNL, the significant target of the released ROS (most probably O_2_^•–^) is the endothelium and NO• released from the endothelium.
